# Fetal weight normograms for singleton pregnancies in a Jordanian population

**DOI:** 10.4103/0256-4947.60519

**Published:** 2010

**Authors:** Nahla Subhi Al-Bayyari, Adel Taha Abu-Heija

**Affiliations:** a From the Department of Applied Sciences, Al-Huson University College, Al-Balqa Applied University, Irbid, Jordan; b From the Department of Obstetrics and Gynecology, Faculty of Medicine, Mutah University, Al-Karak, Jordan

## Abstract

**BACKGROUND AND OBJECTIVES:**

Estimated intrauterine fetal weight (EIUFW) is important for reducing prenatal mortality and morbidity through early detection of faltering growth. Our objectives were to develop patterns of ultrasonically determined EIUFW by gestational age, for normal singleton pregnancies, and to assess the effect of a number of variables on EIUFW.

**METHODS:**

Ultrasonically, EIUFW was obtained from 600 pregnant women who were at 20 to 42 weeks of gestation (WG). EIUFW was categorized into low weight and normal weight using the tenth and twentieth percentile as the cut-off points. Logistic regression was used to calculate the odds ratio and their 95% confidence limits for a number of risk factors hypothesized to be associated with low fetal weight. EIUFW percentiles (twenty-fifth, fiftieth, and seventy-fifth), by gestational age and sex, were calculated for singleton pregnancies.

**RESULTS:**

Up to 32 WG there was no statistically significant difference between male and female fetuses in EIUFW. Between 32 and 39 WG males had significantly (*P*<.05) higher fetal weight than females. Charts of ultrasonically determined EIUFW by gestational age and sex for singleton pregnancies were created. A number of variables were significantly associated with EIUFW such as pregnancy weight gain, maternal hemoglobin level, frequency of antenatal visits, smoking status, and fetal sex.

**CONCLUSION:**

Weight gain during pregnancy should be encouraged for pregnant mothers who gain less than one kilogram per month in the second and third trimester. A prospective study on a national representative sample in Jordan is needed to generate our own standards for fetal growth.

A simple and accurate method of estimating intrauterine fetal weight that can be easily applied to all pregnancies is an important means of reducing prenatal mortality and morbidity through early detection of faltering growth. Several investigators have shown that low estimated fetal weight is associated with high prenatal mortality and morbidity.^1^ Birth weight is a composite of fetal growth and length of gestation, each of which has different contributions and different sequela. Removing the contribution of gestational age to birth weight is the first step in understanding the determinants of fetal growth.^2^ Preterm birth is a prime predictor of neonatal complications, mortality, and developmental delay.^3^ Birth weight may also predict both short- and long-term adverse outcomes. For example, higher birth weight among term infants is associated with birth complications^4^ as well as a reduced risk of cardiovascular disease and hypertension in later life, but an increased risk of obesity.^5^–^8^ In recent years, gains in neonatal survival have been most evident among low birth weight, preterm, and low birth weight (LBW) infants.^9^ Most of the improvements in neonatal survival, since the 1980s, seem to be the consequence of decreasing birth weight-specific mortality rate, which occurred during a period of increasing preterm and LBW rates.^10^

The ultrasound has been used for the examination and evaluation of high-risk pregnancies and for the diagnosis of congenital malformations. During the last two decades ultrasound techniques have been improved and are implemented in most gynecology and obstetric clinics worldwide.^11^ The biophysical profile was used to assess fetal well-being and to confirm fetal gestational age by measuring the biparietal diameter and crown rump length.^12^ Other investigators have predicted intrauterine fetal weight using ultrasonographic measurement of the fetal abdominal circumference.^13^ More recent reports have emphasized the usefulness of this measurement in monitoring normal fetal growth and in detecting intrauterine growth retardation.^14^ All these studies were conducted in Western countries where perinatal medical care and ultrasonographic measurements are advanced.

The ultrasound has been recently introduced for evaluating pregnancies in most Jordanian antenatal clinics and hospitals, but in Jordan, reliable data pertaining to monitoring intrauterine fetal growth and development is lacking. Therefore, research in this area is needed to establish the fetal growth patterns in Jordan and to identify risk factors associated with the occurrence of low intrauterine fetal weight. Such research should be useful for establishing a national comprehensive program for early detection and management of low birth weight fetuses.

## METHODS

This study was undertaken among pregnant women attending the antenatal clinic at Yarmouk University Health Center (YUHC) and Princess Badea Hospital (PBH). The sample in the two clinics was chosen consecutively. The formula used to estimate the sample size was N=μ×sample %, with N=sample size, μ=population size, sample %=0.05 (for population size more than 1000), or N=12000×0.05=600.

A total of 856 pregnant women between 20 and 42 weeks of gestation, who attended the above two clinics for antenatal care or routine follow up were included in the study, and 256 pregnant women with anemia (hemoglobin level < 9.5 g/dL), hypertension (diastolic pressure >90 mm Hg), vaginal bleeding after 20 weeks of gestation, multiple pregnancies and all presentations other than cephalic, were excluded. Our exclusion criteria depended on the obstetric and laboratory test results. Blood pressure was measured for each pregnant woman first and those who were suffering from hypertension (diastolic pressure >90 mm Hg) were excluded from the study.

Data were obtained from 600 pregnant women (300 from women attending YUHC and 300 from women attending PBH) during a visit to the clinic during the study period (June to December 2007). Information on maternal weight in the previous month and the date of last menstrual cycle was obtained from records (confirmed by an obstetrician), from a personal interview using a structured questionnaire that was first pretested and modified, and from ultrasound examinations by four obstetricians (two from each institution), who were not blinded. The ultrasonic measurements included fetal sex, fetal abdominal circumference (AC), and fetal biparietal diameter (BPD). Fetal AC and fetal BPD were used to estimate the intrauterine fetal weight. The BPD measurement was done for each fetus by obtaining a longitudinal section of the fetus by small sliding movements of the transducer on each side of the fetal spine. In this manner, a longitudinal section of the fetal head was obtained, which demonstrated a strong midline echo. By rotating the transducer through 90° a transverse section of the fetal head was obtained. If the section was not the required ovoid shape, the obstetrician made minor rotational adjustments until the shape was correct. If the midline echo was not at the exact center of the section, the obstetrician angled the transducer slightly. This corrected the angle of asynclytism. Slight movements of the transducer up or down the maternal abdomen would locate the correct section, which was then frozen. The BPD was measured by placing the horizontal component of the on-screen calipers on the outer aspects of the echoes from the fetal skull. This had to be at right angles to the midline and at the widest diameter.

Fetal AC is measured by scanning the fetus in a longitudinal plane until the fetal aorta is visualised in its course, through the fetal chest and abdomen. The transducer is then rotated through 90° at the level of the lower end of the fetal ribs. A minor adjustment is then made by moving the transducer up or down the fetal body until the section is obtained. The section is almost a perfect circle, containing only a short section of the umbilical vein in the anterior third of the fetal abdomen. The umbilical vein runs at an angle of approximately 45° through the fetal liver, so if there is more than a short length of vein visible in the section then the section is not at 90° to the spine and the fetal stomach is usually visible as a transonic area on the left side of the section. These parameters are used to predict fetal weight by using the fetal weight estimation table adopted from Shepard et al,^15^ which was built according to this equation BW=−1.7492+0.166 (BPD)+0.046 (AC)−0.002546 (AC) (BPD).

Fetal weight was categorized into two categories: low weight and normal weight using the tenth and twentieth percentile as the cut-off points. Logistic regression was used to calculate the odds ratio and their 95% confidence limits, for a number of risk factors hypothesized to be associated with low fetal weight, after adjustment, for the effect of other variables. The data were examined for interaction by inclusion of interaction terms in the logistic regression models.

Fetal weight was also examined as a continuous variable. The independent effect of a number of variables on the fetal weight was calculated by using multiple linear regressions. The slope (the beta-coefficient) was the amount of change in the dependent (fetal weight) for one unit change in an independent variable, such as, pregnancy weight gain, pre-pregnancy maternal weight, and other variables, after adjusting the fetal gestational age. The Pearson correlation coefficient was used to identify the collinearity between independent variables, when two independent variables were highly correlated; one of these variables was excluded from the regression model. We performed all the analyses using the SPSS statistical package software, version 15 for Windows.

## RESULTS

The comparison between the two samples obtained from PBH and YUHC showed a statistically significant difference between the two antenatal clinics. The differences between the two clinics were probably due to differeneces in the socioeconomic status. Of the 300 pregnant women who attended YUHC, 105 (35%) had a high monthly income (>800 Jordanian dinars, $1128 USD), 145 (48.3%) had a medium income (400-800 JD), and 50 (16.7%) had a low income (<400 JD), while just 25 (8.3%) of the 300 pregnant women who attended PBH had high monthly income, 127 (42.3%) had a medium income and 148 (49.3%) had a low income (*P=*.001). Most of the women who attended YUHC had a higher educational level than the women who attended PBH (*P=*.001).

Several variables were examined for the relationship with fetal weight after adjusting for fetal gestational age and other variables such as fetal sex, pre-pregnancy maternal weight, pregnancy weight gain, and frequency of antenatal visits, in a stepwise multiple linear regression analysis (Table 1). These relationships were also examined for fetal weight below the twentieth percentile using logistic regression, with adjustment for fetal gestational age (Table 2). Fetal sex was not related to the estimated fetal weight when it was included alone with gestational age in a linear regression model (*P* >.05). However, fetal sex was statistically associated with the estimated fetal weight (*P* <.05) in the presence of other variables, namely gestational age, pre-pregnancy maternal weight, pregnancy weight gain, and frequency of antenatal visits, where males had a higher fetal weight than females. In addition, weight gain during pregnancy was categorized into three levels according to Lin and Evans:^16^ low weight gain (gain of 1 kg or less per month in the second or third trimester), medium weight gain (gain of 1 to 2 kg per month in the second or third trimester), and high weight gain (gain of more than 2 kg per month in the second or third trimester) showed a significant association with the estimated fetal weight (*P* <.001) after adjusting for gestational age, pre-pregnancy maternal weight, fetal sex, and frequency of antenatal visits in the multiple linear regression model.

**Table 1:** Step-wise multiple linear regression analysis of the effect of several variables on the estimated fetal weight (each variable is adjusted for all other variables in the table.

Independent variables	Regression coefficients	*P*
Gestational age (weeks)	151.048	.001
Pre-pregnancy maternal weight (kg)	4.509	.001
Fetal sex (male vs. female)	54.146	.02
Pregnancy weight gain (high vs. low)	65.571	.001
Frequency of antenatal visits [less frequent users vs. frequent users (monthly)]	72.256	.001

**Table 2:** Logistic regression analysis of several variables on fetal weight below the 20th percentile versus fetal weight above the 20th percentile.

Variable	Odds ratio	95% CI	*P*
Entire sample (n=600)			
Maternal hemoglobin level (g/dL)			
-Less than 11	1.24	1.01-1.56	.05
-More than 11	1.00		
Frequency of antenatal visits			
-Frequent users (monthly)	1.69	1.20-2.38	.03
-Less frequent users	1.00		
>32 weeks gestation (n=268)			
Maternal hemoglobin level (g/dl)			
-Less than 11	1.98	1.03-3.82	.03
-More than 11	1.00		
Frequency of antenatal visits			
-Frequent users (monthly)	2.49	1.12-5.53	.02
-Less frequent users	1.00		
Smoking			
-Yes	3.42	1.09-10.7	.03
-No	1.00	

Frequent users of antenatal care services had a mean of 72.2 g less weight than less frequent users (*P* <.001). Frequent users were more likely to have low fetal weight fetuses compared to less frequent users (Table 2). For fetuses of greater than 32 weeks of gestation the results were similar. All the women had a hemoglobin level greater than 9.5 g/dL. Hemoglobin levels greater than 9.5 g/dL showed a statistically significant association with estimated fetal weight (*P*<.05). Women with hemoglobin levels < 11 g/dL were more likely to have low weight fetuses compared to women who had hemoglobin levels more than 11 g/dL for the entire sample and for fetuses above 32 weeks gestation (Table 2).

Smoking status was not significantly associated with the estimated fetal weight when using bivariate linear regression analysis (*P* >.05). However, this variable was statistically significant for fetuses above 32 weeks of gestation when comparing those below the twentieth percentile with the remainder (Table 2). Furthermore, pre-pregnancy maternal weight, maternal weight in the previous month of pregnancy, and maternal weight in the existing month of pregnancy were positively associated with the estimated fetal weight.

The same analyses were repeated using the tenth percentile of fetal weight as the cut-off point. None of the factors examined for a relationship with low fetal weight (below the tenth percentile) were statistically significant.

**Figure 1:**
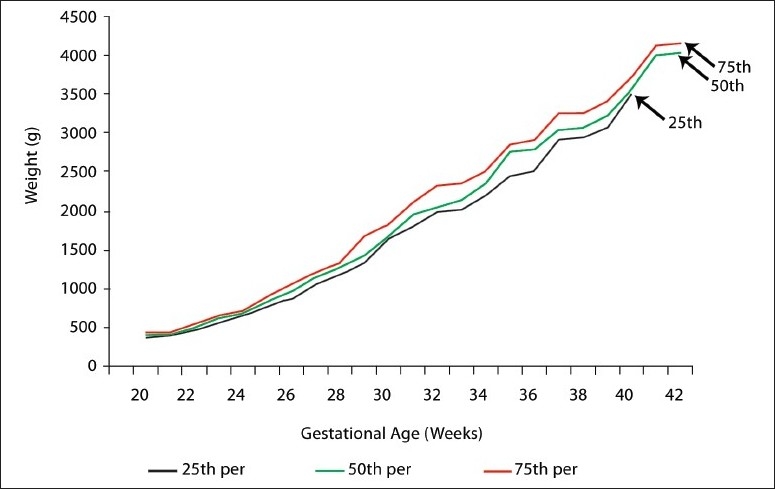
Estimated fetal weight percentiles for (female) singelton pregnancies.

**Figure 2:**
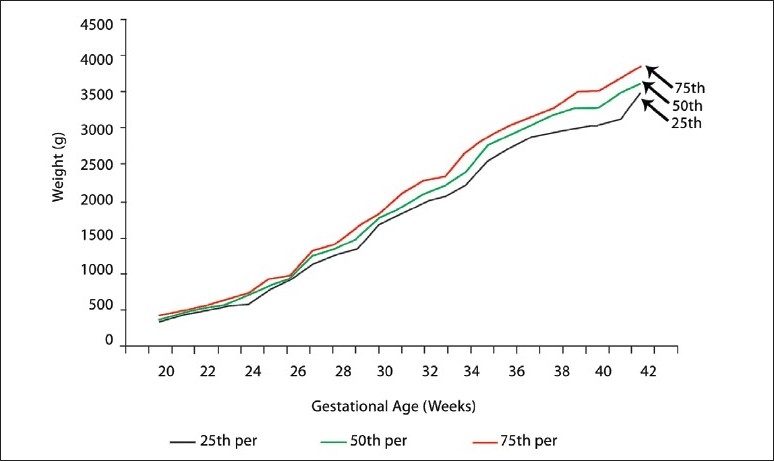
Estimated fetal weight percentiles for (male) singelton pregnancies.

**Figure 3:**
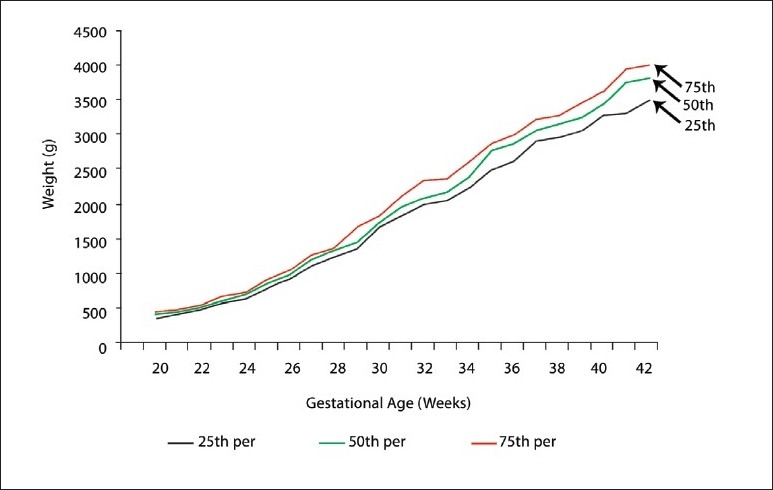
Estimated fetal weight percentiles for (male and female) singelton pregnancies.

The fiftieth, twenty-fifth, and seventy-fifth percentiles of the estimated intrauterine fetal weights at 20 to 42 weeks of gestation are shown for female and male fetuses (Table 3). Up to 32 weeks of gestation there was no statistically significant difference between male and female fetuses. Between 32 and 39 weeks of gestation, males had a higher fetal weight than females (*P* <.05). After 39 weeks of gestation this difference was not statistically significant. The twenty-fifth, fiftieth, and seventy-fifth percentiles of the estimated intrauterine fetal weights throughout the pregnancy from 20 to 42 weeks of gestation are shown for females (Figure 1), males (Figure 2), and for both sexes (Figure 3).

**Table 3:** The fiftieth (median), twenty-fifth and seventy-fifth percentiles of fetal weight in grams for female and male fetuses in the entire sample (n=600).

Week of gestation	No. examined		Female			Male		
	Male	Female	Median	25th percentile	75th percentile	Median	25th percentile	75th percentile
20	8	5	415.0	365.0	427.5	360.0	330.0	417.0
21	6	4	420.0	394.5	450.0	451.0	408.0	483.5
22	9	8	504.0	470.0	545.0	510.0	464.5	536.0
23	11	10	615.0	570.0	662.5	562.0	536.5	637.0
24	13	14	689.0	651.0	716.0	683.0	575.5	710.0
25	9	13	853.0	787.5	906.5	800.0	739.5	909.5
26	18	16	975.0	882.5	1050.0	935.0	902.5	980.0
27	15	19	1160.0	1050.0	1220.0	1240.0	1128.0	1310.0
28	17	18	1280.0	1170.0	1323.5	1335.0	1247.5	1386.0
29	14	15	1420.0	1352.5	1691.5	1488.0	1330.0	1645.0
30	11	20	1677.0	1644.0	1820.0	1770.0	1650.0	1818.5
31	18	15	1961.0	1810.0	2097.5	1895.0	1816.0	2103.5
32	16	19	2060.0	1995.5	2342.5	2100.0	1958.5	2282.0
33	20	17	2130.0	2013.5	2350.0	2206.0	2046.5	2335.0
34	18	15	2360.0	2210.0	2515.0	2380.0	2215.0	2677.0
35	13	17	2750.0	2450.0	2857.0	2770.0	2534.0	2879.5
36	15	13	2790.0	2510.0	2920.0	2920.0	2720.0	3030.0
37	17	15	3050.0	2912.5	3255.0	3060.0	2875.0	3160.0
38	18	13	3065.0	2940.0	3265.0	3200.0	2950.0	3287.5
39	19	21	3220.0	3070.0	3420.0	3265.0	3015.0	3490.0
40	9	10	3555.0	3491.0	3705.0	3270.0	3040.0	3505.0
41	2	2	4000.0	3495.0	4120.5	3500.0	3120.5	3702.5
42	2	3	4020.0	3510.0	4140.0	3611.0	3475.0	3850.0

## DISCUSSION

Our results are consistent with the previous studies that show statistically significant relationships between fetal weight and variables such as maternal weight gain during pregnancy, pre-pregnancy maternal weight, and maternal weight in the previous month of pregnancy, and in the existing month of pregnancy, as well as maternal hemoglobin levels.^17^ Copper et al.^18^ reported that males had a higher fetal weight than females over the entire pregnancy. Others, however, found that this association was present only after 32 weeks of gestation.^19^ In our study, males had a significantly higher fetal weight than females between 32 and 39 weeks of gestation, but after the thirty-ninth week of gestation this association was not significant. This could be due to technical difficulties in assessing the fetal weight after 39 weeks of gestation, when the liquor volume and visibility decreased leading to false positive results. In addition, there was more than one operator involved in the fetal measurements. Frequency of antenatal visits was inversely associated with estimated fetal weight, which was in contrast to the previous studies.^20^ Frequent users of antenatal care were more likely to have low weight fetuses compared to less frequent users. One possible explanation for this unexpected result was that frequent users of antenatal care were more likely to have health problems, and therefore, felt a greater need to receive antenatal care than less frequent users. The results of this study showed a significant association between smoking and fetal weight, but only after 32 weeks of gestation, which agreed with the results of Rantakallo,^21^ while no association was detected for 20 to 42 weeks of gestation. This finding was not consistent with the previous reports, which found that smoking during pregnancy resulted in low birth weight babies.^22^ In the present study, only 5.5% of the mothers were smokers, and many were likely to be light smokers, which may explain the lack of association. Other investigators found a relationship between fetal weight and the intensity of smoking and changes in the pattern of smoking during pregnancy.^23^ Unfortunately, no information was obtained in this study about these two aspects of smoking.

Estimated fetal weight percentiles by gestational age can be used to define the end points of epidemiological studies for factors affecting intrauterine growth or to assess the risk for infant mortality or morbidity. However, the figures presented in the study (Figures 1–3) are not intended to represent intrauterine growth, as the percentiles are based on cross-sectional measurements of estimated fetal weights.

By definition the tenth percentile has commonly been used as the upper limit for defining IUGR (intrauterine growth restriction) in fetuses, in epidemiological studies, but it need not be used to define IUGR for all applications. For epidemiological studies of fetal growth restriction, the fifth or even the tird percentile might be preferable. The percentile selected as most useful may differ between investigators depending on the purpose of the study, the population studied, the medical conditions being evaluated, or the set of intrauterine fetal weights for gestational age standards.^24^ The third, fifth, and tenth percentiles have been used by clinicians to identify infants requiring more intensive observation. As an indicator of infants at the highest risk of poor perinatal outcome, the most appropriate percentile may change with gestational age.^25^ Further study is needed, using linked intrauterine and live-birth infant files together with these new national norms, to determine which percentiles most accurately predict poor perinatal outcome.

Consistent with the previous studies such as that of Arbuckle et al,^26^ our data revealed that the peak of the growth appeared between 30 and 40 weeks of gestation. Growth curves are potentially useful for several purposes in the clinical practice of obstetrics, gynecology, and pediatrics, as well as in public health and social sciences. Adequate fetal growth standards may be used as a basis for more informed clinical judgments and for a better interpretation of results of fetal monitoring. For example, in complicated pregnancies, when evaluating the best time for delivery, determination of the expected fetal weight at the present gestational age and the expected weight gain with each duration of delay may be helpful. Serial determinations of fetal growth by ultrasound and other methods could be used to assess growth restriction and the effects of different interventions. Finally, growth curves may be useful for selecting groups that might benefit from specific public health and educational programs and for evaluating the effects of such programs. The effects of genetic factors, illnesses, and personal habits such as smoking, diet, and addiction on fetal growth, can be evaluated, so that, potential parents can be counseled and effective public health programs can be initiated.

In conclusion, future mothers should be encouraged to have a normal weight before they become pregnant. Also, weight gain during pregnancy should be encouraged for pregnant mothers who gain less than one kilogram per month in the second and third trimesters. Nutrition education programs and food and vitamin supplementation should be provided for women with low hemoglobin. Cigarette smoking should be discouraged for all future mothers. Intrauterine fetal weight charts can be used to evaluate specific nutrition, medical care, educational programs, clinical judgments, and fetal monitoring, and to assess fetal growth restriction. These data should prove useful for researchers investigating the predictors and outcomes of altered fetal growth. A prospective (follow-up) study on a national representative sample in Jordan, or several follow-up studies in different governorates are needed to generate our own standards for fetal growth.
